# Valorization of Soybean Residue (Okara) by Supercritical Carbon Dioxide Extraction: Compositional, Physicochemical, and Functional Properties of Oil and Defatted Powder

**DOI:** 10.3390/foods12142698

**Published:** 2023-07-13

**Authors:** Aunchalee Aussanasuwannakul, Sumitra Boonbumrung, Thidarat Pantoa

**Affiliations:** Department of Food Chemistry and Physics, Institute of Food Research and Product Development, Kasetsart University, Bangkok 10903, Thailand

**Keywords:** supercritical fluid, ethanol, isoflavones, phenolics, antioxidants, tocopherols, fatty acids, rheology, tribology, food ingredient

## Abstract

In the context of food waste valorization, the purpose of this study is to demonstrate the complete valorization of soybean residue (okara) through supercritical carbon dioxide extraction (SCE). Okara oil (OKO) was separated from full-fat powder (FFP) using SCE with and without ethanol (EtOH) as a cosolvent. The kinetics of extraction, chemical composition, and physicochemical, functional, and health-promoting properties of OKO and defatted powder (DFP) were determined. The process yielded 18.5% oil after 450 min. The soluble dietary fiber and protein of the DFP increased significantly; its water and oil absorption capacities increased despite the decrease in swelling capacity corresponding to particle size reduction. The OKO was rich in linoleic and oleic acids, with a ratio of ω6-to-ω3 fatty acids = 9.53, and EtOH increased its phenolic content (0.45 mg GAE/g), aglycone content (239.6 μg/g), and antioxidant capacity (0.195 mg TE/g). The DFP paste showed gel-like consistency and shear-thinning flow behavior, whereas the OKO showed characteristic transition of the product and affected lubrication at contact zones. Both fractions showed potential as food ingredients based on their nutritional and functional properties, as well as the capability of modifying the microstructure of a model food system.

## 1. Introduction

Supercritical carbon dioxide extraction (SCE) is a method that utilizes carbon dioxide in its supercritical state as a solvent to extract various compounds from plant materials. This extraction technique has gained attention due to its ability to be a more environmentally friendly and efficient alternative to traditional solvent-based extraction methods. While SCE is commonly used for extracting essential oils from plants [1], its application in oil extraction from soybean residue or okara is relatively less explored.

Soybean residue, also known as okara, is the byproduct obtained after processing soybeans for the production of soy milk or tofu. Okara contains 5–25% residual oil [2] that can be extracted using different methods, including solvent extraction. SCE has the potential to be used for this purpose as well. While SCE has been recognized as having environmentally friendly benefits and for its selective extraction and minimal thermal degradation, its challenges are high capital costs, complex process optimization, and low oil yields [3].

Studies have demonstrated the feasibility and effectiveness of using SCE for oil extraction from oil seeds. For example, 25% oil yield extracted from flaxseed at 70 °C/55 Mpa [4], 14.4% from grape seeds at 40 °C/25 MPa [5], 92% from sacha inchi seeds at 60 °C/400 bar [6], 17.3% from tomato seeds at 80 °C/55.2 MPa [7], 92.8% from Mexican chia seed at 40–80 °C/450 bar [8], 24.03% from hemp seed at 40 °C/300 bar [9], 16.17% roselle seeds at 40 °C/30 MPa [10], 16.52% canola seeds at 70 °C/18 MPa [11], and 50.03% tea seed at 40 °C/300 bar [12]. These varying reported oil yields depend on the specific conditions and materials used in each study.

Some potential functional benefits and health-promoting properties associated with SCE-extracted oil include high nutritional value, high antioxidant activity, and bioactive compounds. SCE can retain the nutritional composition of the oil, including essential fatty acids, vitamins, and minerals [13], making it a desirable option for obtaining healthy oil extracts. Additionally, many oil seeds contain natural antioxidants, such as phenolic compounds and tocopherols, which can help neutralize harmful free radicals in the body [14]. SCE preserves these antioxidants, enhancing the potential health benefits of the extracted oil [15]. Finally, oil extracts obtained through SCE can contain bioactive compounds, such as sterols, phytosterols, and polyphenols, which may offer various health benefits, such as anti-inflammatory, antimicrobial, and anti-cancer properties [16]. Most studies have indicated that SCE provides superior oil yield, nutrient content, and health-promoting potential compared to conventional extraction methods that use organic solvents, such as hexane.

Likewise, soybean oil has been recognized as a source of heart-healthy polyunsaturated fatty acids [17,18] and vitamin E with antioxidant properties [19], whereas DFP is known as a source of protein, isoflavones, and potential cholesterol-lowering effects [20]. In the context of food waste valorization, however, research that characterizes SCE-extracted oil from okara to support the value of adopting this technology is rather limited. The only study that discussed the use of SCE to extract oil from soybean okara dates to 2006 [21]. Quitain et al. [21] used SCE at 40 °C and 200 bar to recover 25.1% of the OKO, however, without evidencing its potential application. Moreover, as the demand for sustainable food production increases, more recent studies have suggested the potential of using okara powder as an alternative ingredient to a costly soy protein isolate commonly used in the food industry—for example, in meat analogues [22,23]. To the best of the authors’ knowledge, no studies have investigated the quality of oil or the valuable remaining defatted residue obtained from SCE. As demonstrated in other oil seeds, the functional value and health benefits of oil extracted from okara using SCE and DFP, when used as a food ingredient, might outweigh the preconceptual challenges of using SCE with okara. According to the Food and Agriculture Organization of the United Nations (FAO), in 2021, the total world production of soybeans was approximately 372 million tons [24]. Based on this FAO approximation, the global production of okara as a byproduct could be estimated at 446 million tons [25]. This large volume of food waste serves as the rationale for this study. Our study attempted to convert okara into oil and DFP with net zero waste. Recycling this food waste into high-value ingredients would produce an economic impact under the circular economic model.

The aim of this study is to completely valorize okara using environmentally friendly technology and identify potential food applications for the extracts. The objective is to separate oil from okara using SCE and to characterize the compositional, physicochemical, functional, and health-promoting properties of OKO and DFP to define their use as food ingredients.

## 2. Materials and Methods

### 2.1. Materials

The okara used in this study was a byproduct of the Institute of Food Research and Product Development’s pilot soymilk processing line, for which soybean (*Glycine max* L.) was supplied by Doi Kham Food Products Co., Ltd., Bangkok, Thailand. On the same day, fresh okara separated from processing was tray dried at 100 °C for 4 h until its moisture content fell below 10%. The drying conditions used gave the okara an effective moisture diffusivity of (8.67 ± 0.40) × 10^−8^ m^2^/s (n = 3), which was in line with the range of 10^−9^–10^−11^ m^2^/s reported for drying food materials [26] and was comparable to a report for okara using a hot-air oven [27]. The dried residue was then pulverized using a pinmill and sifted using a 60-mesh sieve from Zonytest (Rey and Ronzoni S.A., Buenos Aires, Argentina). The maximum particle size was 272 μm. Three commercial products were selected as benchmarks: (1) okara powder, Sato no Yuki brand (Shikoku Kakoki Co., Ltd., Tokushima, Japan); (2) refined soybean oil for cooking, Angoon brand (Thai Vegetable Oil Public Co., Ltd., Nakorn Pathom, Thailand); and (3) unrefined cold-pressed soybean oil for skin care, Dève Herbes brand (Dève Herbes, New Delhi, India).

### 2.2. Sample Preparation

The dried powder and oil samples were utilized in the analyses without additional preparation, except for rheometry and tribometry. To conduct rheological and tribological tests, the powdered samples were mixed with water to form suspensions of 30% (*w/w*) full-fat powder (FFP) and 10% (*w/w*) defatted powder (DFP). These suspensions were heated to 95 °C with gentle stirring and maintained at that temperature for 30 min. Subsequently, the resulting pastes were cooled to room temperature prior to testing. Additionally, the powdered samples and oil were incorporated into plain yogurt, representing an oil-in-water food matrix, at a concentration of 5% (*w/w*), following the methodologies outlined by [28,29].

### 2.3. Supercritical Fluid Extraction Procedure

The supercritical fluid extraction (SFE) procedure was conducted using a helix SFE system (Applied Separations Inc., Allentown, PA, USA) at the Institute of Food Research and Product Development at Kasetsart University. The system consisted of a solvent and a carbon dioxide pump, a back-pressure regulator, a 1-L extractor vessel with a heating jacket, a pressure transmitter, and a sample collector. The extraction utilized 99.9% pure carbon dioxide (Linde Public Co., Ltd., Samut Prakan, Thailand, and Thonburi Wattana Ltd., Bangkok, Thailand).

To initiate the extraction, 50 g of dry okara flour was placed in a high-pressure stainless steel extractor vessel. The procedure involved a 60-min static extraction followed by a 390-min dynamic extraction at a pressure of 300 bar and a temperature of 50 °C. The flow rate of CO_2_ was maintained at 3 L per minute. Two extraction experiments were conducted: one with ethanol as a cosolvent at a concentration of 25% (*w*/*w*) and one without ethanol. The total extraction time was 450 min. The extracted oils were collected from the sample collector. All experiments were performed in triplicate. The extracted oils and the remaining okara residues were stored at −20 °C for further analysis.

The extraction yield was determined by calculating the ratio of the mass of the crude extract to the total mass of okara and expressing it as a percentage.

### 2.4. Overall Extraction Curves (OEC) and Modeling

The extraction curves were modeled using a spline with three linear segments: the constant extraction rate (CER), falling extraction rate (FER), and diffusion-controlled (DC) periods. R software (version 4.2.2, The R Foundation for Statistical Computing Platform) was used for regression analysis, and Microsoft^®^ Excel for Mac (version 16.69.1, Microsoft) was used to determine the intersection of the two lines.

The spline parameters were employed in the equation proposed by [30] to calculate the extract mass (m_EXT_) or yield at a specific extraction time (t). The equation is given by m_EXT_ = (b_0_ − C_1_b_1_ − C_2_b_2_) + (b_1_ + b_2_ + b_3_)t, where b_1_, b_2_, and b_3_ are linear coefficients of the fitted lines, and C_1_ and C_2_ are intercepts between the first (CER) and second (FER) lines and the second (FER) and third (DC) lines, respectively. C_1_ and C_2_ correspond to the duration of the CER (t_CER_) and FER (t_FER_) periods.

During the CER period (t_CER_), kinetic parameters related to the CER period were calculated: R_CER_ (extract yield, %), M_CER_ (extract mass-transfer rate, kg/s), Y_CER_ (mass ratio of extract in the supercritical phase at the bed outlet, kg extract/kg CO_2_), obtained by dividing M_CER_ by the average solvent flow rate for the CER period [30]. S/F_CER_ (solvent mass to feed mass, kg CO_2_/kg raw material) was determined by multiplying t_CER_ by the average solvent flow rate for the CER period and dividing it by the feed mass.

### 2.5. Chemical Composition

#### 2.5.1. Proximate Composition

The moisture, protein, fat, ash, and total carbohydrate contents of the sample were determined following the methods described in [31]. The contents of total dietary fiber (TDF), insoluble dietary fiber (IDF), and soluble dietary fiber (SDF) in the sample were assayed using the enzymatic-gravimetric method of AOAC 985.29, AOAC 991.42, and AOAC 993.19, respectively, as described in [32].

#### 2.5.2. Lipid Extraction and Fatty Acid Profiles

The dried sample (approximately 2 g) and oil sample (0.1 g) were extracted using a 50 mL chloroform/methanol mixture (2:1), following the methods of [33] with some modifications. The obtained lipid extract was then used to prepare fatty acid methyl esters (FAMEs) according to AOAC (2019) 996.06, 969.33. Gas chromatography with flame ionization detection (Agilent 7890 B, Agilent Technologies, Santa Clara, CA, USA) was employed to determine the fatty acid (FA) profile of the test sample. FAMEs were identified by comparing their retention times with reference standards (Sigma, St. Louis, MO, USA). The FA profile of the samples was expressed as the area percentage of the major FAs.

#### 2.5.3. Acidity (Free Fatty Acid Content, FFA) and Acid Value

The FFA content of the sample was determined using the ISO 660:2020 method [34]. The sample was titrated in ethanol using a standard aqueous sodium hydroxide solution with a phenolphthalein indicator. The FFA content was expressed as a percentage of oleic acid. The acid value of the oil was determined by titrating a solution of the oil in diethyl ether with an alcohol solution of sodium or potassium hydroxide. The acid value was expressed as milligrams of potassium hydroxide required to neutralize the free fatty acids present per gram of oil (mg KOH/g oil).

#### 2.5.4. Peroxide Value (POV)

The POV of the oil was determined by titrating the liberated iodine from potassium iodide with a solution of sodium thiosulfate. The POV was expressed as the reactive oxygen content in milliequivalents (mEq) per kilogram of oil [35].

#### 2.5.5. Total Polyphenol Content (TPC)

The TPC was determined using a colorimetric assay with Folin–Ciocalteu reagent [36]. The reagent was mixed with the sample, and after incubation with Na_2_CO_3_, the absorbance was measured at 765 nm using a UV–visible spectrometer. The TPC was calculated using a gallic acid standard curve and expressed as milligrams equivalent of gallic acid (GAE) per 100 g of dry matter.

#### 2.5.6. Isoflavone

Isoflavone content was determined using high-performance liquid chromatography (HPLC) following the AOAC [37] method. A Shimadzu HPLC system was employed for the qualitative and quantitative analysis of isoflavones in the samples. The HPLC conditions included a 15 × 4.0 mm C18 column, a mobile phase consisting of water, methanol, and glacial acetic acid (88 + 10 + 2 for mobile phase A and 98 + 2 for mobile phase B), and a flow rate of 1 mL/min. Detection was performed at a wavelength of 260 nm, with a column temperature of 50 °C. A 20 μL injection volume and an analysis time of 40 min per sample were used.

#### 2.5.7. Alpha-Tocopherol

Alpha-tocopherol was quantified by HPLC using the following chromatographic conditions: a C18 column, methanol as the mobile phase, a flow rate of 1 mL/min, and detection at a wavelength of 290 nm. The concentration of tocopherol was expressed as alpha-tocopherol (mg/100 g) due to its predominant presence in soybean oils [11].

### 2.6. Physical Properties

#### 2.6.1. Surface Color

Surface color was assessed using a HunterLab XE-Spector colorimeter (Hunter Associates Laboratory, Reston, VA, USA). Color parameters were represented as L*, a*, and b* values. The L* value indicated brightness ranging from white to black, the a* value represented the redness to greenness continuum (positive values for redness and negative values for greenness), and the b* value indicated the yellowness to blueness continuum (positive values for yellowness and negative values for blueness) on the Hunter meter. Prior to testing the samples, the colorimeter was calibrated using a standard white tile (porcelain).

#### 2.6.2. Particle Size Distribution (PSD)

The PSD of okara samples was measured by a Mastersizer laser diffraction particle size analyzer (Mastersizer 3000; Malvern Instruments, Worcester, UK) equipped with the Hydro EV automated wet dispersion unit, and it used 1.45 as a relative refractive index [38]. The mass median diameter represents the averages of three measurements of each sample.

### 2.7. Functional Properties

The functional properties of okara flour are important for the processing and formulation of food products. These properties include water absorption capacity (WAC), oil binding capacity (OBC), and swelling capacity (SC). These functional properties were determined using the methods described by [39] with some modifications.

#### 2.7.1. Water Absorption Capacity (WAC)

The WAC quantifies the maximum amount of water that 1 g of dry material can retain when subjected to an external force in the presence of excess water. To determine the WAC, a 0.5 g sample (w_0_) was weighed in a test tube, followed by the addition of excess water (10 mL). The mixture was shaken for 30 min and subsequently centrifuged at 3000 rpm for 10 min. The supernatant was separated, and the remaining sediment (W_s_) was weighed. The WAC results were expressed as grams of water per gram of sample and calculated as follows:

$$\mathrm{WAC}=(W_{s}-{\;w}_{0})÷w_{0}.$$

#### 2.7.2. Oil Absorption Capacity (OBC)

One gram of the sample (w_0_) was weighed inside a test tube and mixed with 10 mL of vegetable oil (V_1_) using an agitator. The samples were left to rest for 30 min and then centrifuged at 20,000 rpm for 25 min. Immediately after centrifugation, the supernatant was carefully poured into a 10 mL graduated cylinder, and the volume was registered (V_2_). The OBC (milliliters of oil per gram of product) was calculated as follows:

$$\mathrm{OBC}=\left(V_{1}-V_{2}\right)\;÷{\;w}_{0}.$$

#### 2.7.3. Swelling Capacity (SC)

The swelling capacity of a product is its ability to increase its volume in the presence of excess water. In a graduated cylinder, a 2.5 g sample (w_0_) was weighed, and excess water was added (30 mL). The mixture was manually stirred and then left to rest for 24 h at room temperature (27 ± 0.5 °C). The final volume was measured in milliliters (V_f_). The swelling capacity was obtained by applying the following equation:

$$\mathrm{SC}=V_{f}÷{\;w}_{0}.$$

### 2.8. Antioxidant Capacity (AC)

The AC of the sample was assessed using the DPPH radical method described by [40]. A 60 μL sample, diluted 5:100 with methanol, was mixed with 840 μL of DPPH-adjusted solution. After incubation at room temperature, the absorbance of the mixture was measured at 517 nm using a UV–visible spectrometer. The absorbance resulting from the reaction between the DPPH radical solution and the sample was used to calculate the percentage inhibition of DPPH. A calibration curve was constructed using Trolox as a standard (6-hydroxy-2,5,7,8-tetramethylchromane-2-carboxylic acid). Finally, the AC of the sample was expressed as μmol of the Trolox equivalent (TE) per 100 g of dry matter (d.m.).

### 2.9. Rheological and Tribological Properties

#### 2.9.1. Rheometry

The steady rotational shear measurements of the viscosity and flow behaviors of the samples followed those previously reported by [41] using a modular compact rheometer (Model MCR 302; Anton Paar, Graz, Austria) with parallel-plate geometry, a 25-mm diameter for the upper plate, and a gap width of 1 mm. The measurements were performed at 4 °C and 37 °C, which are the average storage and body temperatures, respectively. The shear rate was applied within the range of 0.1–1000 s^−1^ with 100 measuring points using ascending logarithmic steps. Viscosity at a shear rate of 50 s^−1^ was used as a predictor for thickness perception. The recorded shear rate vs. the shear stress data was fitted to the Herschel–Bukley model, from which the yield stress (Pa), flow behavior index (n, dimensionless), and consistency index (Pa.s*^n^*) were derived. Data were recorded and analyzed using RheoCompass software (Version 1.30, Anton Paar GmbH, Graz, Austria).

#### 2.9.2. Tribometry

**Figure 1:**
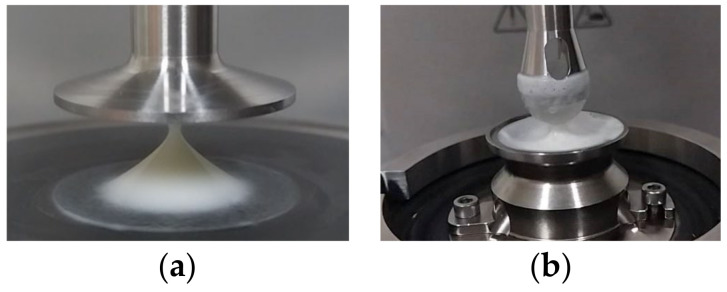
Detailed view of (**a**) the geometry used in rheometry and (**b**) the configuration used in tribometry.

The lubrication properties of the samples were measured using the MCR 302 rheometer with a ball-on-three-pin configuration according to [31]. For each test, around 3 g of the sample was gently loaded into the sample holder and spread out to cover the three stationary polydimethylsiloxane (PDMS) pins fixed into it. Measurements were performed at 37 °C with a constant normal force of 1 N. The friction coefficients between the rotating 0.5-in soda-lime glass ball and the three stationary, cylindrical PDMS pins were recorded to measure their rotational speeds, ranging from 0.01 to 1000 mm/s, using RheoCompass software (Figure 1).

### 2.10. Statistical Analysis

Statistical analysis was obtained via analysis of variance (ANOVA), followed by Tukey’s test using R software [42]. The results, expressed as mean ± standard deviation, were considered statistically significant if *p* ≤ 0.05. The analyses were replicated at least three times.

## 3. Results and Discussion

### 3.1. Extraction

The efficiency of SFE for soybean oil extraction is influenced by various factors, including pressure, temperature, modifier, water content, and solvent flow rate [43]. Table 1 provides a comparison of SCE studies conducted on different raw materials for soybean oil extraction. The highest oil yield is achieved within the pressure range of 200–350 bar and the temperature range of 35–50 °C. The oil yield from full-fat raw materials typically falls between 16% and 25% due to the primary factors of extraction pressure and temperature. In our study, utilizing extraction conditions of 300 bar and 50 °C, an extraction yield of 18.5% was obtained, which aligns with the range reported by other researchers.

When comparing equal temperatures (e.g., 40 °C), lower pressure levels tend to result in higher yields (Table 1). In our preliminary investigation, we observed a positive effect of extraction pressure (300 bar vs. 400 bar) on yield, while extraction temperature (40 °C vs. 50 °C) did not significantly affect the yield (*p >* 0.05). Similarly, in the optimization of key extraction parameters using a quadratic model, only pressure was found to have a significant effect on increasing soybean oil yield [48]. These findings contradict the observations of [44]. Additionally, Rodríguez-Ruiz et al. [49] noted in hydroalcoholic extraction that increasing the temperature enhanced the extracted amounts by improving the solubility of bioactive compounds through modifications in solvent properties.

**Figure 2:**
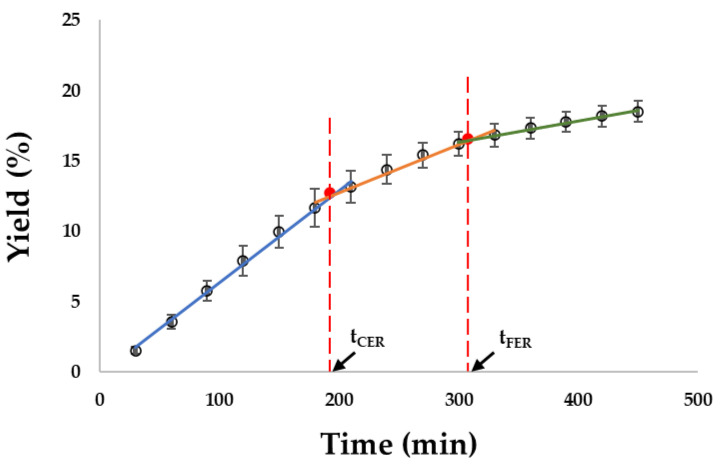
Overall extraction curve for okara + CO_2_ obtained at 300 bar, 50 °C. Blue line represents the extraction during the constant extraction rate (CER) period, orange line is during the falling extraction rate (FER) period, and green line is during the diffusion-controlled (DC) rate period.

**Figure 3:**
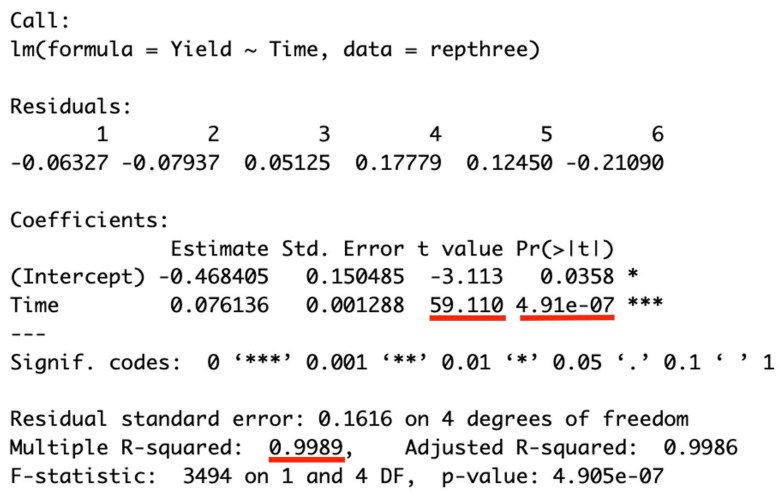
R output of a simple linear regression of extraction yield on extraction time during the constant extraction rate (CER) period.

Extraction kinetics play a crucial role in assessing the efficiency and sustainability of SFE processes [30]. In this study, the global yield of the extract was estimated by fitting the overall extraction curve (OEC) using a spline model. Figure 2 displays the kinetic curve, and the data-fitting results are presented in Figure 3. For the constant extraction rate (CER) period (30–180 min) of the SCE conditions (Figure 2, blue line), a linear regression model was applied to predict the extraction yield. The model exhibited a significant relationship, with a t value of 59.11 and a corresponding *p* value < 0, indicating its reliability (Figure 3). The coefficient of determination (R^2^) of 0.9989 suggests that 99.89% of the variation in extraction yield can be explained by this model. Similarly, the data fitting for the falling extraction rate (FER) period (210–300 min) and diffusion-controlled (DC) rate period (330–450 min) yielded R^2^ values of 0.9891 and 0.9963, respectively, indicating the adequacy of the models for these periods.

Upon constructing the spline model using the three straight lines, the key kinetic parameters were estimated. The triplicate runs of the SCE conditions demonstrated the repeatability of the extraction curve, with coefficient of variation (CV) values of 3.94, 0.35, and 3.52 for t_CER_, t_FER_, and m_EXT_, respectively, indicating low variability among the triplicate extractions.

Figure 2 displays the extraction kinetics of okara using supercritical fluid extraction (SCE) at lab scales. The curves exhibit three distinct periods commonly observed in the extraction of natural products with supercritical fluids: CER period, FER period (transition), and DC rate period. The total yield obtained from okara extraction was 18.5% over a 450-min duration. In a previous study by Quitain et al. [21], an extraction recovery of 63.5% for okara oil (OKO) was reported using 200 bar pressure, 10% ethanol concentration, and 40 °C temperature. The extraction process involved a 2-h holding period followed by continuous flow extraction for 5 h. Table 2 presents the adjusted kinetic parameters based on the obtained overall extraction curves (OECs) for SCE + okara.

The CER period primarily involves the extraction of solutes present on the surface of solid substratum particles or released from disrupted cells during processing [30]. The solutes extracted during this period are readily accessible, and mass transfer in the external film near the particle surface is governed by convection [50]. For the SCE + okara process, the CER period is characterized by a mass transfer rate (M_CER_) of 19 × 10^−7^ kg/s, a duration (t_CER_) of 193 min, a yield (R_CER_) of 11.7% *w*/*w*, and a mass ratio of solute in the fluid phase at the outlet of the extractor vessel (Y_CER_) of 3.36 × 10^−5^ kg oil/kg CO_2_. These kinetic parameters for the SCE + okara process are comparable to those observed for cloves using a 290 mL extraction vessel (in contrast to the 1 L extractor vessel used for okara SCE) operated at 150 bar and 40 °C.

During the CER period, the yield achieved (R_CER_) accounted for 63% of the total yield, whereas cloves reported 52% (Table 2). In the case of herbs, spices, and edible fungi, careful pretreatment enables extraction of 70–90% of soluble material during the CER period [51]. The variation in oil yield, both R_CER_ and total yield (R_total_), can be attributed to differences in raw material quality. As highlighted by [52], the cellulosic structure of vegetable material influences extract solubility in CO_2_, which is defined as a pseudo-ternary system. However, SCE + okara constitutes a more complex system with solutes (such as fatty acids, triglycerides, and tocopherols) exhibiting different solubilities in CO_2_, thus resembling a quaternary system. The values of M_CER_ (mass transfer rate) and Y_CER_ (mass ratio of solute in the fluid phase) were two orders of magnitude lower for SCE + okara compared to cloves, suggesting that the SCE process for okara is slower, possibly due to solubility limitations of the multicomponent mixture in supercritical CO_2_ and the inert solid nature of okara’s microstructure.

**Table 2:** Kinetic parameters of the SCE process for extracting oil from okara (current study) compared to cloves [53].

Parameter *	SCE300/50 + Okara	SCE150/40 + Clove
t_CER_ (min)	193 ± 7.59	42
t_FER_ (min)	308 ± 1.08	145
M_CER_ (kg/s)	(19 ± 1) × 10^−7^	58.7 × 10^−7^
Y_CER_ (kg oil/kg CO_2_)	(3.36 ± 1.18) × 10^−5^	61.2 × 10^−3^
R_CER_ (%, g/g)	11.7 ± 1.0	7.74
R_total_ (%, g/g)	18.5 ± 0.8	14.9

* Mean of triplicate analyses; t_CER_—constant extraction rate period; t_FER_—falling extraction rate period; M_CER_—mass-transfer rate during CER period; Y_CER_—mass ratio of solute in the supercritical phase at bed outlet during CER period; R_CER_—yield achieved during CER period; R_total_—total yield.

The CER period for SCE + okara lasted only 193 min out of the total 450-min extraction time. The lipid extraction process was primarily diffusion-limited, with internal mass transfer being the rate-controlling step in the SFE of lipid molecules [54]. In subcritical fluid extraction using a water + EtOH mixture, lower mass flow rates resulted in increased extraction amounts due to longer contact times between the solvent and the plant material [49].

### 3.2. Compositional, Physicochemical, and Functional Properties of DFP

**Figure 4:**
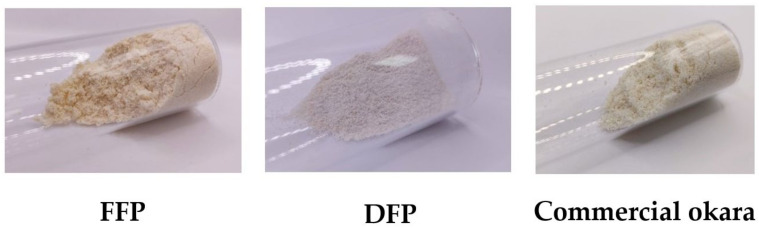
Raw material powder for SCE (FFP), okara powder after SCE at 300 bar, 50 °C (DFP), and commercial okara powder.

A dried FFP (tray dried at 100 °C) was used as raw material for SCE, and the residue after lipid extraction was labeled as DFP. The external appearance of the raw material and residue is different, as shown in Figure 4. The DFP sample appeared granular, while the FFP appeared bulkier and fluffier. The DFP (0.24% fat) also appeared redder (a* = 82.0) and yellower (b* = 74.4) compared to the FFP (2.88% fat), with a* = 3.37 and b* = 20.3 (Table 3).

Table 3 shows the chemical composition of fresh okara, FFP, oil extracted from okara using SCE, and commercial okara powder. The SCE process increased the soluble dietary fiber of the dry samples from 0.38% to 1.82%. A significant increase in protein from 35% in FFP to 44% in DFP was also observed. This concentrated protein of the FFP sample after SCE was even higher than the commercial benchmark (20%). These differences in the appearance, surface color, and composition of okara powder samples suggested the effect of fat removal on the microstructure of okara powder.

**Figure 5:**
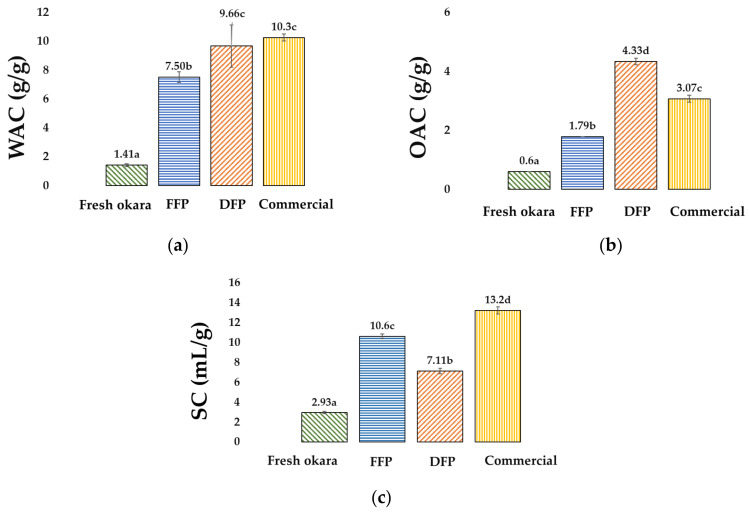
Functional properties of fresh okara, FFP, DFP, and commercial benchmarks: (**a**) water absorption capacity (WAC, g/g); (**b**) oil absorption capacity (OAC, g/g); (**c**) swelling capacity (SC, mL/g). ^a–d^ Means with different letters within the same response are different (*p* < 0.05; n = 3).

The functional properties of okara provide information about interactions between okara and water, predicting the processing and mass formation, as well as its possible behavior in other food matrices. Our functional properties for FFP (Figure 5) were in close range to those of [39] using similar convection drying. Drying generally improves the functional properties of fresh okara. Our observed improved WAC, OAC, and SC in FFP are in line with [55] and could possibly be explained by the improved surface hydrophobicity of the protein.

The DFP had an increase in WAC and OAC values over FFP (9.66 g/g vs. 7.50 g/g and 4.33 g/g vs. 1.79, respectively; *p <* 0.05); this was in contrast to a decrease in SC of DFP at 7.11 mL/g as compared to FFP (10.6 mL/g) and commercial benchmark (13.2 mL/g). Although its SC was not as good as the commercial benchmark, the DFP exhibited a comparable ability to absorb water (Figure 5). Removing fat from FFP with SCE could concentrate its protein content from 36% to 44%; however, the SC decreased by 33%. Our result is contrary to others who have reported improved swelling capacity after being defatted of flour from other fiber sources (amaranth, [56]; guar, [57]; coconut, [58]; walnut, [59]). The microstructure of the insoluble fractions of defatted soy flour high in the content of polysaccharides and proteins can be characterized as carbohydrates covalently attached to proteins in a glycoprotein matrix (arabino–galactan–proteins and glucomannan–proteins [60]). With such a dense glycoprotein matrix, the swelling of crude fiber could hardly occur after absorbing water and expanding the volume of the flour further. The capacity of water absorption and swelling thickness of the hybrid composites containing sugar palm fiber are directly related to the density, the presence of voids, and the bond between the fiber and the matrix; composites showed decreased swelling with increasing content of the natural fiber [61]. Another explanation for the decrease in SC might be heat treatment during the SFE process, which affects the surface charge and rate of protein hydration. Our DFP, nonetheless, has superior protein content, hydration, and oil-binding capacity to those reported in defatted amaranth flour [62].

Size reduction of the dried powder using a pin mill decreased the particle size of okara from 586.27 μm to 272.33 μm. Defatting using SCE further decreased the particle size of okara to 238 μm (Table 3). The size of the okara powder samples (from biggest to smallest: fresh > FFP > DFP) is inversely related to their WAC (from lower to higher: fresh < FFP < DFP).

In line with our okara powder, with a relatively high proportion of insoluble fiber (44.8%), is rice husk, with the major constituents of 38% alpha cellulose, 22% lignin, 20% ash, and 19% silica. The smaller the particle size, the smaller the water absorption index, and the larger the water solubility and smaller swelling power values [63]. The effect of particle size on solubility and dispersibility is limited to a certain size range, and a surface area that is too large (small particles) can lead to poor dispersibility of a powder [38]. According to [38], a moderate increase in particle size (from 15 to 24 μm) improved powder dispersibility of spray-dried soy powders. The underlying basis of the different functional properties under the size range is probably differences in particle morphology affected by SCE. From the segregation of rosehip seeds in fractions with different oil contents, larger particles (≥0.85 mm) were mainly composed of tough, lignified testa fragments devoid of oil, whereas smaller particles (≤0.425 mm) contained mostly brittle, oil-rich germ fragments [64].

However, in this study, SCE might have affected the microstructure of the powder differently. According to [65], the morphology of the SCE-treated plum kernel protein isolate (protein ~35%) was characterized by reduced particle size. Loose and tiny fragments with greater homogeneity in particle-size distribution resulted from homogenization induced by carbon dioxide under a supercritical state at higher temperatures (60 °C).

### 3.3. Compositional, Physicochemical, and Functional Characterization of OKO

#### 3.3.1. Appearance and Color

**Figure 6:**
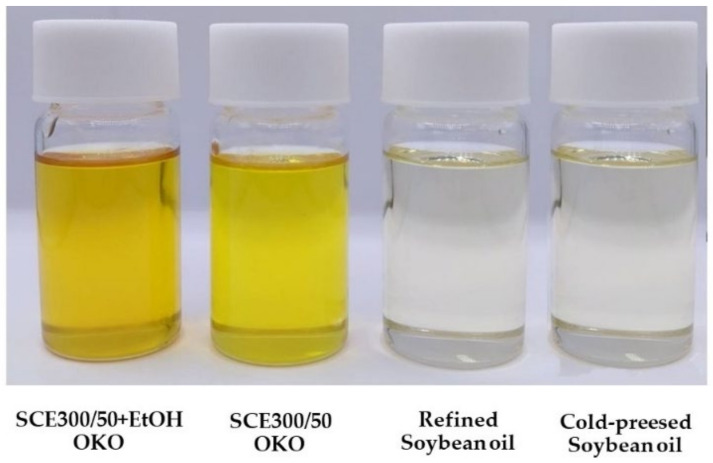
OKO after SCE at 300 bar 50 °C with and without EtOH, commercial refined soybean oil, and commercial unrefined cold-pressed soybean oil.

Figure 6 and Table 4 show the appearance and display color values of OKO. The SCE + EtOH oil had the highest redness (a*) and yellowness (b*) values, while the highest lightness (L*) values were cooking and skincare soybean oil. Vegetable oils vary in color from a light greenish yellow through canary or amber yellow to brown or deep red, and contain varying proportions of several different coloring matters commonly found in the tissues of plants [66]. In the conventional process of soybean oil extraction, undesirably colored impurities are removed by bleaching with an adsorptive reagent [67]. Our SCE OKO color was contrary to those reported by [47], who found that soybean oil extracted with SCE is lighter in color compared to hexane-extracted crude oil from the same beans, probably because of different raw materials. The increased redness and yellowness of the oil extracts compared to the commercial benchmarks suggest the presence of phytonutrients and pigments enriched by SCE and SCE with EtOH processes.

#### 3.3.2. Fatty Acid Composition

Table 4 presents the fatty acid (FA) composition of soybean oils obtained from various sources: fresh okara, supercritical extracted OKO, and commercial soybean oils. In fresh okara, the dominant FA is linoleic acid (49%), followed by oleic acid (24.2%), palmitic acid (10.5%), linolenic acid (5.18%), and stearic acid (4.15%). This FA pattern is consistent in both SCE OKO and commercial soybean oils. Other FAs, such as myristic acid, heptadecanoic acid, arachidic acid, behenic acid, lignoceric acid, palmitoleic acid, and eicosenoic acid, are present at much lower levels (<0.5%) in all three matrices. Our FA profile differs significantly from that reported for soybean oil obtained from different soybean varieties using n-hexane as the solvent. In a previous study, the fatty acid composition included linoleic acid (49–53%), oleic acid (22.6–24%), palmitic acid (11–13.5%), linolenic acid (6.5–8%), and stearic acid (3.02–4.9%) [68]. This variation in the FA profile is attributed to the specific genetic characteristics of the soybean varieties rather than to the extraction methods employed.

The ω − 6/ω − 3 ratio is commonly used to evaluate the balance of essential fatty acids (FA) in the diet [69]. Seed oils typically exhibit a high ω − 6/ω − 3 ratio, as reported by others (e.g., 7.2 by [70]), and it is expected to eventually align with the recommended ratio suggested by the FAO [71] when consumed as part of a healthy diet. According to the FAO [72], there is no specific recommendation for the ω − 6 to ω − 3 ratio or the LA (linoleic acid) to ALA (alpha-linolenic acid) ratio as long as the intakes of ω − 6 and ω − 3 fatty acids fall within the recommended range of 0.5–2% and 2.5–9% of energy, respectively. In our study, the ω − 6/ω − 3 ratio of SCE OKO was slightly higher than that of fresh okara (8 vs. 7; *p* < 0.05), suggesting a possible modification of essential fatty acids during the thermal and SCE treatment.

Regarding the PUFA (polyunsaturated fatty acid)/SFA (saturated fatty acid) ratio, OKO exhibited a high PUFA content (55%) and a lower SFA content (16%) in our study, falling within the range reported elsewhere. The proportion of PUFA in OKO decreased compared to that in the initial okara (63%), resulting in a mean PUFA/SFA ratio of 3.43 for OKO and 4.13 for okara. This indicates a slight increase in the percentage of SFA during the elaboration process, primarily due to palmitic and stearic acids.

A similar trend was observed in soybean oil obtained via supercritical fluid extraction (100 °C, 7500 psi or 517 bar, 3 L/m) with CO_2_ [33]. The predominant fatty acids in soybean and soybean products, except for soybean oil, were linoleic acid (C18:2ω − 6) at 47.57%, oleic acid (C18:1) at 16.02%, and linolenic acid (C18:3ω − 3) at 12.11% *w*/*w*, resulting in a ω − 6/ω − 3 ratio of 3.92 and a PUFA/SFA ratio of 2.49.

#### 3.3.3. Isoflavones, Total Phenolic Compounds, and Antioxidant Capacity

Increased total phenolic compounds (TPC), isoflavone content, and antioxidant capacity were observed in SCE OKO when EtOH was used as a cosolvent (Table 4). This finding aligns with a previous study [44], where oil extracts from soybean residues obtained through SCE at 35 °C, 40 MPa with the addition of EtOH (25% *w*/*w*) exhibited the best antioxidant properties. The TPC values ranged from 10.6 to 16.0 mg GAE/100 g d.m., flavonoid content ranged from 65.0 to 31.3 QE/100 g d.m., and antioxidant capacity measured by DPPH values ranged from 9.7 to 12.0 μmol TE/100 g d.m. EtOH has been widely used to enhance the solubility of polyphenols and antioxidants in subcritical fluid extraction of oil from soybean meal [49] and soybean seed coat [73]. The concentration of total phenolic compounds strongly depends on the ethanol concentration.

In our study, we investigated the two major forms of isoflavones (IFs) present in okara: aglycones (daidzein, glycitein, and genistein) and β-glucosides (daidzin, glycitin, and genistin). Supercritical fluid extraction (CO_2_) has been employed for OKO recovery and was able to extract approximately 180 μg of genistein and daidzein per gram of extract using the following parameters: 30 Mpa, 40 °C, and 5% ethanol as a cosolvent [21]. In our study, we detected IFs in both the oil fraction and the remaining powder matrix. In the oil fraction, SCE could only detect daidzin, whereas SCE + EtOH detected aglycones (daidzein, glycitein, and genistein) at a concentration of 239.6 μg/g oil (Table 4). The concentration of aglycones increased, while the concentration of β-glucosides decreased in the okara powder as the degree of processing increased, from fresh to FFP and to DFP (Table 4). This change is likely due to hydrolysis during the processing steps involving heating and grinding. The SCE extraction resulted in higher concentrations of β-glucosides (42.51 mg/100g DW) and aglycones (772.5 μg/g DW) in the powder.

Supercritical carbon dioxide is a comparable, if not superior, solvent compared to other studied solvents, such as water and short-chain alcohols. For example, using a 70:30 ethanol and water solution at 20 °C, β-glucosides and aglycones were recovered at 357 μg/g and 230 μg/g dry extract, respectively, whereas using water alone, β-glucosides and aglycones were recovered at 198 μg/g and 33 μg/g dry extract [74]. Subcritical water extraction yielded β-glucosides at 891.1 μg/g and aglycones at 47.4 μg/g dry okara in 5 min under the conditions of 3.98 Mpa pressure, 146 °C temperature, and a solid-to-solvent ratio of 1:20 (*w*/*v*) [75].

Our results, supported by a recent review by [76], suggest that okara is a promising source of isoflavones with various recovery methods and solvents. However, multiple stages of extraction and pretreatment of the solid matrix are necessary to enhance the solubility of these compounds in the extraction solvent.

#### 3.3.4. Alpha-Tocopherol, Peroxide Value, Acid Value, and Free Fatty Acids

Alpha-tocopherol, which has been found to positively correlate with the oxidative stability of vegetable oils [77], was not detected in any of the samples in the present study (Table 4). Soybean oil has a significant amount of tocopherol present in the oil [78]. Using SCE at 31.0 MPa and 90 °C at the top to 70 °C at the bottom of the extractor, Chang et al. [79] could recover 83.6% of tocopherols from soybean deodorizer distillate. However, soybean oil generally has a large degree of unsaturation of fatty acids that significantly diminishes its stability [80]. SCE OKO are oxidatively less stable relative to the refined and cold pressed soybean oil, as indicated by their higher AV and FFA; this can be explained by their corresponding higher proportion of polyunsaturated fatty acids (Table 4). Commercial refined soybean oil showed the highest PV, whereas AV and FFV were not detected. This might be explained by the study by [81], who found that hexane-extracted degummed soybean oils exhibit better oxidative stability than the SCE of soybean oil at 50 °C and 8000 psi because phosphatides, which are natural antioxidants, are essentially absent in SCE-extracted oils. It is also worth noting that the oxidative stability values of the commercial samples are approximate, as they can vary depending on different raw materials, processing processes, storage times, and conditions. Generally, the PV, AV, and FFA of all samples are within acceptable values for soybean oil at 10 m Eq O_2_/kg, 0.6 mg KOH/g, and 0.1–0.5%, respectively [82].

### 3.4. Rheometry and Tribometry of Okara Paste and Oil

**Figure 7:**
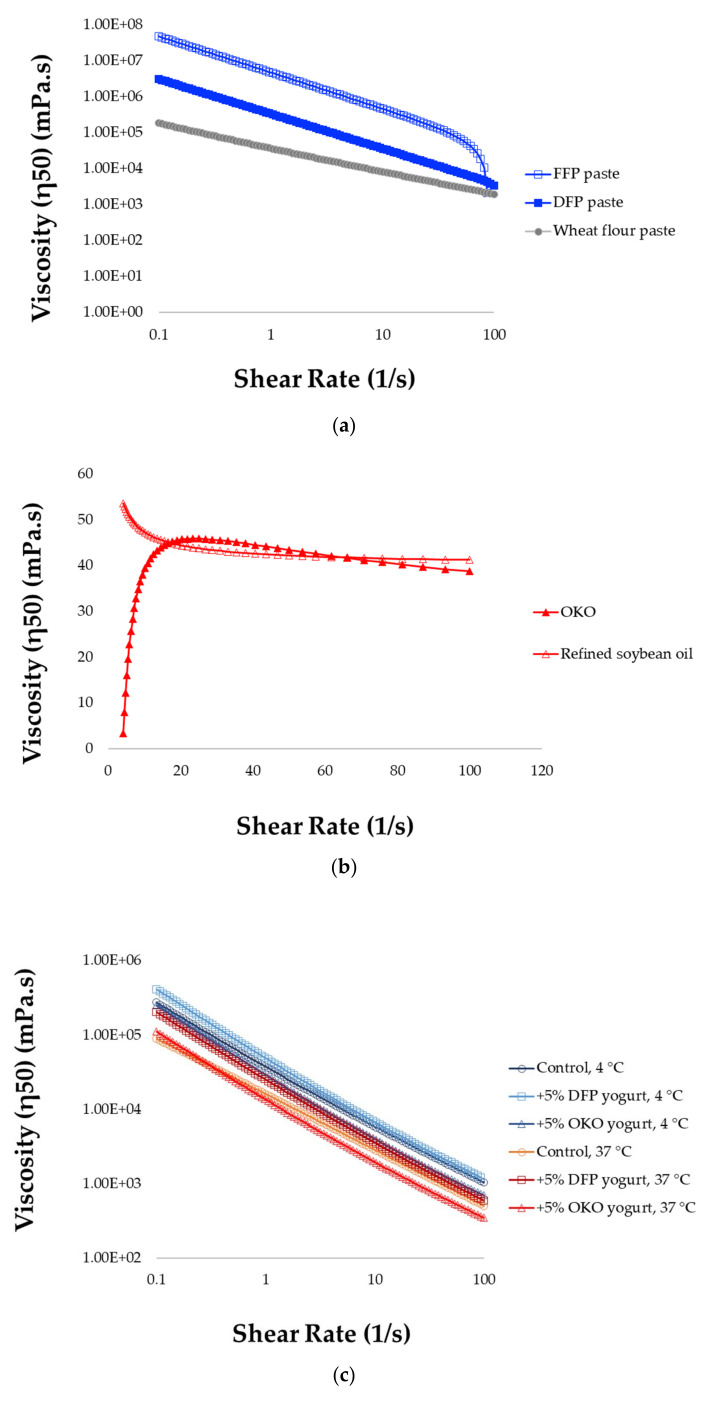
Viscosity curve of (**a**) okara paste and (**b**) okara oil compared with commercial benchmarks and (**c**) yogurt fortified with okara extracts.

Cooked paste was prepared from okara powder for rheological and tribological analyses. In line with its better water absorption capacity, its homogenous paste of the DFP can be attained at a relatively lower concentration at 10% (suggesting better protein–water interaction) compared to the FFP attained at 30%. Jamalullail et al. [83] reported that the lowest gelation concentration (LGC) of DFP improved from 18 g/100 mL of whole soybean flour to 14 g/100 mL, responding to the rise of protein and carbohydrate contents, which induced more intense intermolecular contacts from protein gelation and starch gelatinization. Better water retention and gelation of DFP demonstrated its potential as an ingredient in colloidal food system formulations, such as yogurt-like products. Okara extracts were both analyzed as is or in gelled form under simulated conditions relevant to their applications. Oscillatory shear measurement suggested that all the paste samples exhibited gel-like consistency at the low-shear range (shear rate < 0.1 s^−1^), with the DFP exhibiting greater rigidity (higher G′ value) and larger deformation amplitude (LVE range) than the FFP and wheat paste at 0.5%, 0.1%, and 0.05%, respectively. Under rotational shear, okara paste exhibited shear-thinning flow behavior with viscosity at 50 s^−1^ ($\eta_{50}$) of FFP paste superior to the DFP one (6.82 × 10^4^ vs. 2.95 × 10^3^ Pa.s, *p* < 0.05; Figure 7a). However, at a higher shear rate above 100 s^−1^, the FFP seemed to lose stability and exhibited complex flow, shown as a non-linear curve and lack of fit (R^2^ = 0.36). Under a static shear rate, $\eta_{50}$ of OKO and commercial cooking oil are identical (*p* < 0.05) at 4.49 × 10^1^ Pa.s (Figure 7b). Below 20 s^−1^, however, the viscosity of both oils depends on shear rates; this indicates a range of non-Newtonian behavior, and that oils may be classified as Bingham fluids.

Adding 5% okara extracts to yogurt maintained the flow characteristic (shear-thinning) of the product (Figure 7c). Yogurt fortified with OKO compared with DFP paste showed slightly lower viscosity ($\eta_{50}$; 5.88 × 10^2^ vs. 1.26 × 10^3^ Pa.s) and a lower consistency index (*K*; 3.88 vs. 11.5 Pa.s*^n^*) at 37 °C. The interaction effect of temperature by fortification on $\eta_{50}$ is significant (*p* = 0.046); refrigeration generally increased the flow properties of yogurt, and at each temperature, yogurt-fortified DFP was the thickest.

**Figure 8:**
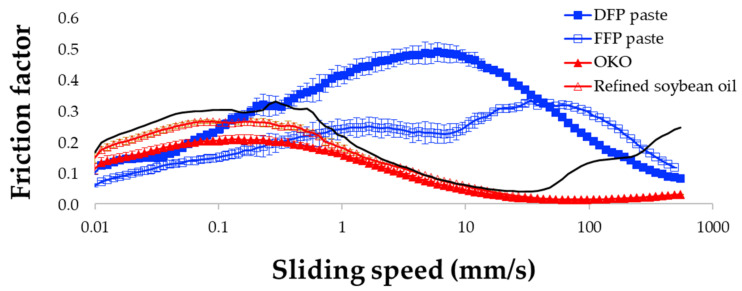
Friction profile of DFP and FFP paste, OKO, and commercially refined soybean oil.

As cooked paste, with a higher content of lipid and fiber, the higher lipid content of 30% FFP paste showed a friction curve with a flatter shape, with a lower friction value (μ) than the 10% DFP (Figure 8). This pattern in the FFP is similar to that reported by [84] in soybean paste, showing a flat plateau profile and relatively low friction (0.01–550 mm/s); according to the authors, this typical profile for soybean flour is attributed to its protein and lipid (protein = 34.98%, lipid = 16.29%, fiber = 15.49%). The single curve profile of the 10% DFP (protein = 44%, lipid = 0.24%, fiber = 46.6%), where μ peaked at 5 mm/s, could be explained by its higher protein content, which further inflated the effect of defatting on surface lubrication, compared to the FFP (protein = 35.5%, lipid = 2.88%, fiber = 52.71%), with lower μ observed between 0.1 and 10 mm/s.

The effect of supercritical fluid extraction (SFE) on protein quality has been reported in the literature mostly in a positive way, for example, by Yu et al. [85], and there is no direct link between the altered quality and tribological properties. At temperatures well below the denaturation temperatures of soy protein, SFE did not cause denaturation of protein, but rather resulted in superior functional properties [86]. However, SFE could have induced aggregation of okara proteins like those reported for mackerel proteins [87]. Large aggregated protein particles cause jamming of the contact area and increase friction [88]. The effect of protein concentration on tribology has also been reported. The soy protein isolate at a relatively high concentration (9–12% vs. 6%) that showed an increase of μ at sliding speed 1–10 mm/s, similar to our study, was caused by particle jamming and free particle rolling [89].

OKO showed lower μ than both paste samples above the dry regime (1 mm/s; Figure 8). While OKO droplets can easily enter and lubricate contact zones at an early speed range, okara paste, which primarily consists of large insoluble polysaccharides and protein matrices, requires a much higher shear rate to break down such complex structures. The fact that μ was in the range of 1.77 × 10^−2^ and 2.13 × 10^−1^, slightly lower than in the commercial sample (1.85 × 10^−2^ to 2.69 × 10^−1^), can be explained by their different fatty acid compositions. The lower μ of OKO, with higher percentages of oleic acid (24.2%) and lower percentages of linoleic acid (20.7%) than commercial cooking oil at 20.7% and 51.5%, respectively, is a result of the structure of the fatty acids [90].

The viscosity and flow behavior of yogurt were affected differently between 5% DFP and 5% oil fortification (Figure 7c). Both samples exhibited shear-thinning behavior, with apparent viscosity decreasing with increasing shear rates. At 37 °C, the apparent viscosity at 50 s^−1^ ($\eta_{50}$) and the consistency index (*K*) were affected by the 5% DFP more significantly when compared to the control and 5% oil. As expected, both viscosity and yield stress increased for all samples at refrigeration temperature (4 °C), and the discrimination between fortification approaches was less pronounced. The greater rheological stability of fiber-rich and protein-rich DFP could be explained by its superior water-immobilizing ability. Its higher apparent viscosity is also an indication of a higher-thickness sensation.

**Figure 9:**
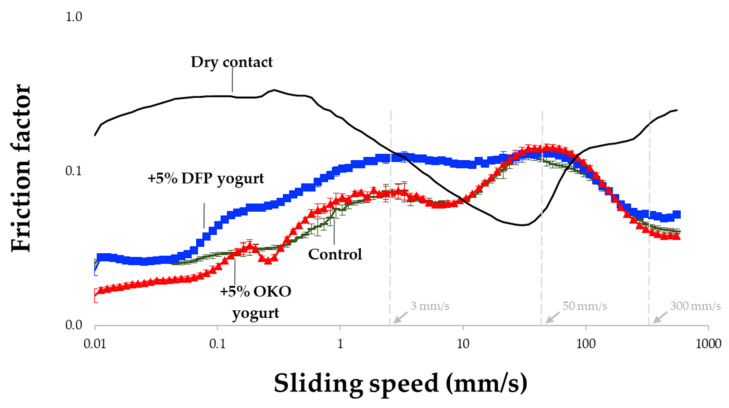
Friction profile of yogurt fortified with okara extracts.

The friction curve of yogurt can be divided into four zones, with transitions occurring around velocities of approximately 3, 50, and 300 mm/s (Figure 9), which is consistent with previous findings [91]. Incorporating DFP into the yogurt led to higher μ and more noticeable differences compared to the control sample in the first two zones (up to 50 mm/s), corresponding to the dry and boundary regimes in the conventional Stribeck curve. This increase in friction can be attributed to the high water and oil absorption capacity of DFP, which may have prevented soluble substances and lightly dispersed particles in the liquid whey from spreading and covering the PDMS pins.

As free fat globules migrated from the gel matrix, and fluid gel started to enter the contact zone, their lubricating and load-bearing effects resulted in a slight decrease in friction at 10 mm/s, followed by a continuous rise to the maximum μ at 50 mm/s. According to [92], water released during high strain and increasing sliding speed in fiber-enriched yogurt contributes to increased friction and is associated with a more compact texture and enhanced particulate mouthfeel. Fiber plays a unique role in optimizing the textural properties of yogurt by affecting the stability of oil-in-water emulsions, as the lubricating properties of such emulsions largely depend on the stabilization of oil droplets. For instance, fiber in the form of microgel particles, particularly when stabilized by soy protein and fat, has been observed to modulate creaminess in yogurt [93]. Given that both fiber and protein possess lubricating properties, the presence of these key components in DFP suggests its potential as a microstructure-modulating or stabilizing agent in yogurt-like products.

The addition of 5% OKO did not significantly affect the friction profile of yogurt compared to the control sample. This could be attributed to the larger droplet size of OKO, which is immiscible with the continuous liquid phase and thus unable to enter the contact zone. Consequently, the tribological properties of the continuous phase, consisting of whey protein and free fat globules, determined the overall friction behavior. However, at higher shear rates (around 500 mm/s), the contribution of OKO to surface lubrication became evident as smaller oil droplets formed a load-bearing fluid film and eliminated the viscous drag that separates the surfaces. Similar observations were made in a previous study involving a thickened sauce model [41], emphasizing the significance of shear-thinning behavior in the hydrodynamic friction regime, where friction is influenced by rheological properties at higher shear rates.

Our findings align with the pattern observed in plant-based emulsions and support the proposed mechanism, where increasing rotation speed allows smaller oil droplets to enter the gap between surfaces, creating a rolling layer and reducing friction. As seen in soy-based yogurt, the tribology of emulsions highly depends on how oil droplets are stabilized, indicating the potential of using okara powder and oil to adjust the lubricating properties and microstructure in yogurt formulations. Although 5% OKO had minimal impact on the rheological properties of yogurt, except for a threefold reduction in the consistency index (*K*), it shows promise as a functional oil additive for modifying mouthfeel and providing nutritional enrichment in yogurt-like products.

While we have demonstrated the potential of DFP and OKO as food ingredients through rheo- and tribometry-based analyses, their effects on sensory perception remain unknown. Further research is needed to explore the sensory aspects of these ingredients.

## 4. Conclusions

Supercritical carbon dioxide extraction (SCE) is an eco-friendly method for extracting compounds from plants, including okara, a soybean residue. This study extracted and characterized okara oil (OKO) and defatted okara powder (DFP) for potential food applications, contributing to sustainable food production. Okara SCE exhibited a repeatable extraction curve with a total yield of 18.5%. Differences in appearance, color, and composition were observed between the DFP and FFP. SCE improved the water absorption and oil-binding capacity of DFP but decreased its swelling capacity. OKO contained phytonutrients, pigments, and major fatty acids, such as linoleic, oleic, palmitic, linolenic, and stearic acids. The omega-6/omega-3 ratio was slightly higher in OKO than the initial okara, indicating possible fatty acid modifications during processing. SCE with ethanol increased the total phenolic compounds, isoflavone content, and antioxidant capacity in OKO. Alpha-tocopherol was not detected in any samples. OKO had a higher peroxide value, acid value, and free fatty acids compared to refined and cold-pressed soybean oil due to its higher proportion of polyunsaturated fatty acids. Okara paste exhibited better water absorption and gelation properties compared to full-fat okara paste. OKO showed lower friction values than okara paste and commercial cooking oil, with lubricating properties at higher shear rates. Both extracts showed potential as food ingredients, but further research is needed on their sensory perception and process optimization for increased yield and bioactive compounds. The economic and environmental viability of SCE for okara valorization should also be explored for industrial applications. In the context of food waste valorization, this study has demonstrated a complete use of okara through SCE, showing an extraction process and extracts with qualities comparable to the industrial benchmarks.

## Figures and Tables

**Table 1:** Yield of oil separated from different matrices using SCE.

Condition		Matrix	Yield (wt%) *	References
CO_2_ Pressure	Temperature			
300 bar	50 °C	Okara	18.5 ± 0.8	Current study
400 bar	35 °C	Soymeal	2–3.5	[44]
200 bar	40 °C	Okara	25.1 ± 1.9	[21]
300 bar	40 °C	Soybean	19.5	[45]
300 bar	40 °C	Soybean	16.4	[46]
350 bar	50 °C	Soybean	19.9	[47]

* Mean value ± standard deviation (only those reported by the authors are shown; n ≥ 3).

**Table 3:** Physicochemical and compositional properties of fresh okara, FFP, DFP, and commercial benchmarks.

	Fresh Okara	FFP	DFP	Commercial
**Color**				
L*	-	83.0 ± 3.3 ^b^	78.3 ± 3.1 ^a^	86.4 ± 3.5 ^c^
a*	-	3.37 ± 0.14 ^b^	82.0 ± 3.3 ^c^	0.46 ± 0.02 ^a^
b*	-	20.3 ± 0.8 ^b^	74.4 ± 3.0 ^c^	15.8 ± 0.6 ^a^
**Composition**				
Moisture %	79.2 ± 3.2 ^c^	3.82 ± 0.15 ^a^	3.21 ± 0.13 ^a^	7.56 ± 0.30 ^b^
Protein %	30.7 ± 1.2 ^b^	35.5 ± 1.4 ^c^	44.0 ± 1.8 ^d^	19.6 ± 0.8 ^a^
Fat %	20.8 ± 0.8 ^d^	2.88 ± 0.12 ^b^	0.24 ± 0.01 ^a^	4.84 ± 0.19 ^c^
Ash %	3.75 ± 0.15 ^a^	3.03 ± 0.12 ^a^	3.61 ± 0.14 ^a^	3.94 ± 0.15 ^a^
Carbohydrate %	44.8 ± 1.8 ^a^	54.8 ± 2.2 ^c^	49.0 ± 2.0 ^b^	67.2 ± 2.7 ^d^
Total dietary fiber (g/100 g)	44.7 ± 1.8 ^a^	52.7 ± 2.1 ^c^	46.6 ± 1.9 ^b^	63.3 ± 2.5 ^d^
Soluble dietary fiber (g/100 g)	4.18 ± 0.16 ^d^	0.38 ± 0.02 ^a^	1.82 ± 0.07 ^b^	3.32 ± 0.13 ^c^
Insoluble dietary fiber (g/100 g)	40.5 ± 1.6 ^a^	52.3 ± 2.1 ^c^	44.8 ± 1.8 ^b^	60.0 ± 2.4 ^d^
**Particle size (μm)**	586 ± 18 ^d^	272 ± 2 ^b^	238 ± 7 ^a^	386 ± 4 ^c^

^a–d^ Means with different letters within the same response are different (*p* < 0.05; n = 3).

**Table 4:** Physicochemical and compositional properties of fresh okara, SCE OKO with and without EtOH, commercial refined soybean oil, and commercial unrefined cold-pressed soybean oil.

	Fresh Okara	SCE300/50 OKO	SCE300/50 + EtOHOKO	Refined Soybean Oil	Cold-Pressed Soybean Oil
**Color**					
L*	-	80.3 ± 0.6 ^b^	73.2 ± 0.6 ^a^	98.6 ± 0.8 ^d^	95.0 ± 0.8 ^c^
a*	-	0.06 ± 0.00 ^a^	7.03 ± 0.06 ^d^	3.52 ± 0.03 ^b^	4.46 ± 0.04 ^c^
b*	-	96.9 ± 0.8 ^c^	99.3 ± 0.8 ^d^	14.5 ± 0.1 ^a^	15.9 ± 0.1 ^b^
**Fatty acid** ^1^					
Palmitic (C16:0)	10.7 ± 0.1 ^a^	11.1 ± 0.1 ^b^	11.4 ± 0.1 ^b^	10.5 ± 0.1 ^a^	10.4 ± 0.1 ^a^
Stearic (C18:0)	3.56 ± 0.03 ^a^	4.34 ± 0.04 ^b^	4.21 ± 0.03 ^b^	4.15 ± 0.03 ^b^	4.58 ± 0.04 ^b^
Oleic (C18:1)	19.6 ± 0.2 ^a^	20.7 ± 0.2 ^b^	20.1 ± 0.2 ^b^	24.2 ± 0.2 ^c^	20.7 ± 0.2 ^b^
Linoleic (C18:2 ω − 6)	55.1 ± 0.4 ^c^	51.5 ± 0.4 ^c^	52.1 ± 0.4 ^bc^	49.4 ± 0.4 ^a^	52.3 ± 0.4 ^bc^
Linolenic (C18:3 ω − 3)	7.67 ± 0.06 ^c^	6.34 ± 0.05 ^b^	6.35 ± 0.05 ^b^	5.18 ± 0.04 ^a^	5.30 ± 0.04 ^a^
%SFA	15.2 ± 0.1 ^a^	16.4 ± 0.1 ^b^	16.4 ± 0.1 ^b^	15.9 ± 0.1 ^a^	16.0 ± 0.1 ^ab^
%MUFA	21.2 ± 0.2 ^ab^	20.9 ± 0.2 ^a^	20.3 ± 0.2 ^a^	24.8 ± 0.2 ^b^	21.6 ± 0.2 ^ab^
%PUFA	62.8 ± 0.5 ^d^	57.9 ± 0.5 ^b^	58.5 ± 0.5 ^c^	54.6 ± 0.4 ^a^	57.6 ± 0.5 ^b^
PUFA/SFA	4.13 ± 0.03 ^c^	3.53 ± 0.03 ^b^	3.56 ± 0.03 ^b^	3.43 ± 0.03 ^a^	3.60 ± 0.03 ^a^
ω − 6/ω − 3	7.18 ± 0.06 ^a^	8.13 ± 0.07 ^b^	8.21 ± 0.07 ^b^	9.53 ± 0.08 ^c^	9.87 ± 0.08 ^c^
**Chemical compounds/activity**					
Alpha-Tocopherol	-	N.D. ^2^	N.D.	N.D.	N.D.
Total Isoflavone	77.7 ± 0.6 ^d^	3.29 ± 0.03 ^b^	24.0 ± 0.2 ^c^	2.53 ± 0.02 ^a^	N.D.
Daidzin	3.85 ± 0.03 ^b^	3.29 ± 0.03 ^b^	N.D.	2.53 ± 0.02 ^a^	N.D.
Glycitin	3.51 ± 0.03	N.D.	N.D.	N.D.	N.D.
Genistin	10.1 ± 0.1	N.D.	N.D.	N.D.	N.D.
Daidzein	18.1 ± 0.2 ^b^	N.D.	7.33 ± 0.06 ^a^	N.D.	N.D.
Glycitein	5.05 ± 0.04 ^a^	N.D.	4.45 ± 0.04 ^a^	N.D.	N.D.
Genistein	37.1 ± 0.3 ^b^	N.D.	12.2 ± 0.1 ^a^	N.D.	N.D.
Total phenolic compounds (GAE/g DW)	1.93 ± 0.02 ^c^	0.10 ± 0.00 ^ab^	0.45 ± 0.00 ^b^	0.05 ± 0.00 ^a^	0.05 ± 0.00 ^a^
Antioxidant capacity (μmol TE/100 g d.m.)	0.12 ± 0.00 ^ab^	0.1 ± 0.00 ^ab^	0.20 ± 0.00 ^b^	0.08 ± 0.00 ^a^	0.08 ± 0.00 ^a^
Free fatty acid	-	1.12 ± 0.00 ^b^	1.43 ± 0.01 ^b^	N.D.	0.16 ± 0.00 ^a^
Acid value	-	2.58 ± 0.02 ^b^	2.89 ± 0.02 ^b^	N.D.	0.33 ± 0.00 ^a^
Peroxide value	-	5.88 ± 0.05 ^b^	4.77 ± 0.04 ^a^	9.87 ± 0.08 ^d^	7.82 ± 0.06 ^c^

^1^ Reported as mean % abundance of total fatty acids; ^2^ N.D. = Not detected; ^a–d^ Means with different letters within the same response are different (*p* < 0.05; n = 3).

## Data Availability

All related data and methods are presented in this paper. Additional inquiries should be addressed to the corresponding author.
